# The immune dysregulation of fibrosis: insights into immune-fibrotic crosstalk and potential therapeutic targets

**DOI:** 10.3389/fimmu.2026.1770516

**Published:** 2026-03-11

**Authors:** Hua-Xin Kang, Yi-Fei Fu, Hao Wu, Xie-Lin Yan, Jun-Jie Wu, Ling-Zhen Jiang, Lei-Sheng Wang, Ya-Ru Xiao, Zhen-Zhu Zhang, Feng-Lai Yuan, Zhi-Tong Zuo

**Affiliations:** 1 Affiliated Hospital of Jiangnan University, Wuxi, China; 2 Wuxi Medical College, Jiangnan University, Wuxi, China; 3 Tongren Hospital Shanghai Jiao Tong University School of Medicine, Shanghai, China; 4 Donghai County People’s Hospital, Lianyungang, China

**Keywords:** immune cells, immune dysregulation, myofibroblast, pharmacotherapy, pulmonary fibrosis, therapeutic target

## Abstract

Pulmonary fibrosis is the converging pathological outcome of chronic inflammatory lung diseases with diverse etiologies. Sustained inflammation disrupts immune cell homeostasis, driving aberrant activation and differentiation of myofibroblasts and leading to excessive extracellular matrix deposition within the lung interstitium. Emerging evidence indicates that immune dysregulation contributes to myofibroblast activation, and that immunomodulatory therapies may reverse fibrotic progression across diverse disease models. In this Review, we dissect the mechanisms by which immune dysregulation promotes fibrosis in distinct pathological contexts, highlighting the heterogeneity of immune–fibrotic interactions. We further discuss emerging targets with potential for precision intervention, aiming to inform the development of adjunctive and personalized therapeutic strategies.

## 1 Introduction

Pulmonary fibrosis represents the final common pathway of acute and chronic lung injuries, characterized by irreversible structural remodeling and progressive impairment of pulmonary function (1). In advanced stages, widespread parenchymal distortion disrupts alveolar architecture and severely compromises gas exchange, eventually leading to respiratory failure, pulmonary hypertension, and other severe complications (2). Currently available antifibrotic agents provide limited benefit in treating pulmonary fibrosis and are often associated with significant side effects (3). Lung transplantation remains the only curative option, but its broader utilization is constrained by donor shortage and immunological complications. Consequently, median survival following diagnosis remains below three years (4). The global burden of pulmonary fibrosis continues to grow, accelerated by increased viral infections, such as SARS-CoV-2, and an aging population, emphasizing its escalating public health significance. The severe situation of pulmonary fibrosis urgently demands a deeper understanding of its pathogenesis, as only by clarifying the mechanisms can we bring new hope for treatment.

The pathogenesis of pulmonary fibrosis is a highly orchestrated, dynamic process that involves multiple players. Myofibroblasts are recognized as key effectors driving disease progression (5). It is widely accepted that myofibroblast activation is regulated by various factors, including epithelial injury, mechanical stretch, and cytokines. Once activated, myofibroblasts secrete large amounts of excessive extracellular matrix (ECM), particularly collagen. This excessive ECM deposition thickens and stiffens the lung parenchyma, reducing tissue elasticity and impairing pulmonary function (5). Notably, insights gained from the COVID-19 pandemic suggest persistent immune activation in some patients, even after pathogen clearance, implicating ongoing immune dysregulation—not merely initial injury—as critical in driving fibrotic progression (6). The lung, connected to the external environment via the airways, contains a large number of immune cells to combat pathogens and harmful substances. In response to injury, neutrophils, macrophages, dendritic cells (DCs), and Natural killer (NK) cells are activated to clear pathogens through phagocytosis, production of reactive oxygen species (ROS), and neutrophil extracellular traps (NETs) (7). Persistent inflammation leads to macrophage polarization imbalance and T cell subset alterations. Immune homeostasis is disrupted, creating a chronic pro-inflammatory and profibrotic immune microenvironment. In this context, immune cells continuously release profibrotic factors, such as transforming growth factor-β (TGF-β), IL-13, and IL-6 (8, 9), activating signaling pathways like TGF-β/Smad and Wnt/β-catenin to drive myofibroblast activation and accelerate fibrosis (8).

Although antifibrotic agents such as pirfenidone and nintedanib suppress myofibroblast activity directly (10), emerging evidence suggests they may also modulate immune responses, thereby indirectly attenuating fibrotic progression (11). Meanwhile, targeted immunotherapies such as tocilizumab, rituximab (12), Bruton’s tyrosine kinase (BTK) inhibitors (13), and host defense peptide derivatives like RP-832c (14) have shown promising antifibrotic effects by correcting immune dysregulation. These agents act through pathways involving B cell suppression, IL-6 blockade, and inhibition of TGF-β signaling. By contrast, in idiopathic pulmonary fibrosis (IPF), conventional immunosuppressive regimens have failed to confer clinical benefit and have, in some settings, been associated with adverse outcomes. This divergence suggests that immune involvement in pulmonary fibrosis cannot be adequately addressed through non-selective suppression, but instead depends on context-specific modulation of immune pathways. In this Review, we examine the immune–fibrotic interface in pulmonary fibrosis, with particular attention to cellular and molecular interactions between immune cells and myofibroblasts. We further discuss how immune contributions vary across distinct etiologies and consider emerging immunotherapeutic strategies that may enable more precise, mechanism-informed intervention. This review integrates immune cell heterogeneity, signaling network crosstalk, and disease-stage specificity into a unified framework.

## 2 Role of immune cells in the microenvironment of pulmonary fibrosis

**Figure 1:**
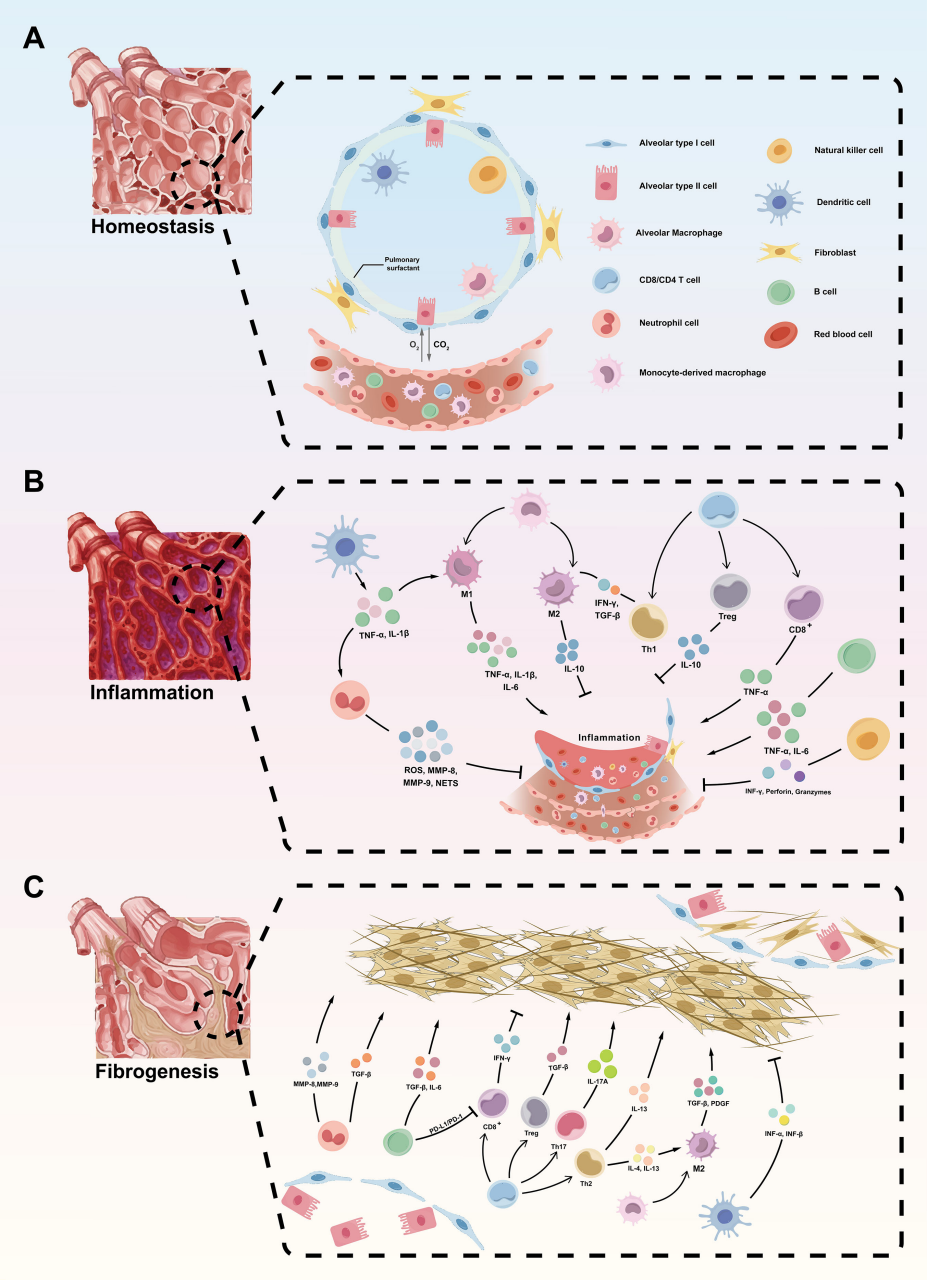
Cellular and molecular mechanisms of immune microenvironment underlying the progression from pulmonary homeostasis to fibrosis. **(A)** Homeostasis: Under normal physiological conditions, the alveolar microenvironment is sustained by a well-orchestrated network of epithelial cells (alveolar type I and II cells), resident immune cells (including alveolar macrophages, dendritic cells (DCs), and Natural killer (NK) cells, and structural cells such as fibroblasts. Alveolar type II cells secrete pulmonary surfactant and facilitate epithelial repair, while resident macrophages maintain immune homeostasis. **(B)** Inflammation: In response to injury or pathogenic stimuli, both innate and adaptive immune responses are activated. Neutrophils, monocyte-derived macrophages (MDMs), and DCs release pro-inflammatory cytokines (e.g., TNF-α, IL-1β, IL-6) and mediators such as reactive oxygen species (ROS) and matrix metalloproteinases (MMPs), exacerbating tissue damage. Activated T cells (e.g., Th1, CD8^+^) and M1 macrophages further sustain the inflammatory response, while regulatory T cells (Tregs) and M2 macrophages counteract inflammation via IL-10 and transforming growth factor-β (TGF-β). **(C)** Fibrogenesis: Chronic inflammation promotes dysregulated tissue repair and excessive extracellular matrix (ECM) deposition. Profibrotic cytokines (e.g., TGF-β, IL-4, IL-13) drive fibroblast activation and differentiation into myofibroblasts, leading to progressive fibrotic remodeling. Multiple immune cell subsets, including Th2, Th17, CD8^+^ T cells, and M2 macrophages, contribute to fibrosis through cytokine-mediated pathways (e.g., TGF-β, PDGF). Disruption of epithelial integrity and an imbalanced immune microenvironment further perpetuate fibrogenesis.

The pulmonary immune microenvironment functions as a dynamic regulatory interface that integrates tissue injury with inflammatory responses and fibrotic remodeling during the development of pulmonary fibrosis (15). Collectively, dynamic crosstalk between injured epithelial cells and infiltrating or resident immune cells constitutes a central organizing axis of this microenvironment, shaping both inflammatory persistence and subsequent fibroblast activation. In response to exogenous insults or endogenous cellular damage, lung tissues release damage-associated molecular patterns (DAMPs) and pathogen-associated molecular patterns (PAMPs), thereby initiating innate immune activation and promoting the rapid recruitment of inflammatory cells (16). Activated immune cells further amplify this response through the production of pro-inflammatory cytokines, which facilitate additional immune cell recruitment and intensify local inflammatory signaling (9). Persistent monocyte infiltration and antigen presentation subsequently engage adaptive immune responses, driving the expansion and functional polarization of lymphocytes and sustaining immune activation within the pulmonary microenvironment (17). Fibroblasts additionally function as immunoregulatory sentinels, sensing tissue damage and secreting mediators that modulate early immune responses and coordinate initial repair processes (18). Timely resolution of inflammation facilitates phenotypic transitions in immune cells toward anti-inflammatory and reparative states, restoring fibroblast quiescence and enabling tissue regeneration (19). Conversely, sustained or unresolved injury disrupts this regulatory equilibrium, leading to chronic immune activation and prolonged engagement of both innate and adaptive immune compartments (20). Under these circumstances, immune cells continually produce profibrotic mediators, which drive fibroblast-to-myofibroblast transdifferentiation (21). These resultant myofibroblasts exhibit enhanced contractile properties and ECM production, culminating in excessive ECM deposition, structural distortion, and impaired pulmonary function (**Figure 1**).

Viewed across disease progression, immune regulation in pulmonary fibrosis unfolds along a temporally and functionally coordinated continuum, encompassing the maintenance of alveolar homeostasis, immune-driven epithelial injury during inflammatory responses, and the persistence of immune signaling that supports fibrotic remodeling.

### 2.1 Immune maintenance of alveolar epithelial homeostasis

In the healthy lung, alveolar architecture and function are maintained within an immune niche dominated by resident immune populations, including alveolar macrophages (AMs) and DCs (22). These cells persist in a largely quiescent state, supporting efficient gas exchange while mediating the routine clearance of inhaled particles and senescent cells (23). Under these conditions, immune activity remains tightly constrained, fibroblasts retain a resting phenotype, and tissue integrity is preserved without progression to overt inflammatory or fibrotic responses.

### 2.2 Immune-driven epithelial injury and maladaptive repair during inflammation

Following acute lung injury, rapid innate immune activation imposes substantial stress on alveolar epithelial cells (AECs), resulting in early disruption of epithelial integrity and compromise of barrier homeostasis. Disruption of this tightly regulated immune equilibrium represents the earliest step toward fibrotic remodeling. Among the first responders, neutrophils are swiftly recruited into the injured alveolar space through IL-8–mediated engagement of CXCR1/2, which activates downstream PI3K/Akt and JAK/STAT signaling to enhance chemotaxis, survival, and effector capacity (24–26). At sites of epithelial damage, activated neutrophils release a broad spectrum of cytotoxic mediators, including neutrophil elastase (NE) (27), matrix metalloproteinases (MMP-8 and MMP-9), and ROS, collectively exacerbating oxidative injury to AECs and weakening alveolar barrier integrity (28). In parallel, excessive ROS activates mitogen-activated protein kinase (MAPK) signaling within the alveolar niche, further amplifying inflammatory stress and reinforcing epithelial susceptibility to injury (27).

Beyond these direct cytotoxic effects, NET formation represents a pivotal mechanism linking acute innate immune activation to epithelial dysfunction. Although NETs participate in pathogen containment, their histone-rich components induce apoptosis of AECs and concurrently activate platelets to release TGF-β, thereby initiating profibrotic signaling cascades at sites of epithelial injury (29, 30). This early rise in TGF-β not only perturbs epithelial repair programs but also establishes a microenvironment conducive to fibroblast activation and oxidative stress, creating conditions that favor maladaptive tissue remodeling (31). Moreover, activation of the TGF-β/Smad axis prolongs neutrophil survival within injured lungs, further sustaining inflammatory pressure on the alveolar epithelium during the acute phase (32, 33).

Concomitantly, circulating monocytes are recruited to injured lungs and differentiate toward a classically activated M1 macrophage phenotype. These macrophages secrete high levels of pro-inflammatory cytokines, including TNF-α, IL-1β, and IL-6, thereby reinforcing epithelial inflammation and perpetuating the recruitment of additional innate immune cells into the alveolar compartment (34, 35). TNF-α and IL-1β directly impair epithelial homeostasis by promoting inflammatory signaling within AECs, whereas IL-6 contributes to the amplification and persistence of local cytokine networks. In parallel, macrophage-derived chemokines such as CXCL9 and CXCL10 facilitate continued neutrophil influx, further intensifying epithelial injury during acute inflammation (35–37).

DCs simultaneously integrate epithelial-derived danger signals and participate in shaping the evolving immune–epithelial interface. DAMPs released from injured AECs and surrounding stromal cells, particularly high-mobility group box 1 (HMGB1), activate DCs through Toll-like receptor signaling, driving DC maturation characterized by upregulation of MHC-class II (MHC-II) and costimulatory molecules (38). Activated DCs subsequently secrete pro-inflammatory cytokines such as TNF-α and IL-1β, further amplifying chemokine expression and sustaining recruitment of neutrophils and macrophages into the alveolar microenvironment (39, 40). This feed-forward inflammatory circuitry interferes with effective epithelial regeneration and prolongs epithelial stress responses during acute injury.

Although adaptive immune responses emerge more gradually, early T cell–derived signals already contribute to shaping epithelial fate. Th1-derived IFN-γ may transiently counterbalance profibrotic signaling by suppressing TGF-β/Smad activation and limiting fibroblast expansion, thereby indirectly supporting epithelial preservation (41). However, in the context of persistent inflammatory cues, ongoing epithelial damage, and sustained innate immune activation, these protective effects are progressively overridden. CD8^+^ T cells further contribute to epithelial injury through release of TNF-α, which promotes AEC apoptosis and exacerbates alveolar damage during sustained inflammation (42). Collectively, the convergence of neutrophil-derived cytotoxic mediators, macrophage-driven inflammatory amplification, DC-mediated immune integration, and early T cell–derived signals establishes a hostile alveolar microenvironment in which epithelial repair is impaired, inflammatory stress is sustained, and profibrotic pathways are primed, thereby biasing tissue responses toward maladaptive repair and subsequent fibrotic progression. Collectively, these immune-driven processes sustain epithelial injury, compromise regenerative capacity, and create a tissue context in which profibrotic signaling can emerge.

### 2.3 Immune reprogramming and fibroblast activation during fibrogenesis

When inflammatory responses fail to resolve and epithelial injury persists, the pulmonary immune microenvironment is progressively reprogrammed toward a profibrotic state. This shift is accompanied by persistent epithelial dysfunction and sustained activation of mesenchymal cells, resulting in fibroblast-dominated tissue remodeling in which M2-polarized macrophages assume a central regulatory role.

Under chronic injury conditions, alternatively activated macrophages acquire a profibrotic phenotype characterized by sustained production of TGF-β, platelet-derived growth factor (PDGF), connective tissue growth factor (CTGF), vascular endothelial growth factor (VEGF), and activin A (43, 44). These mediators promote fibroblast proliferation, myofibroblast differentiation, and ECM deposition. Macrophage-derived tissue inhibitors of metalloproteinases (TIMP-1) further suppress ECM degradation and favor matrix accumulation (45). Macrophages in fibrotic lungs may also adopt osteoclast-like properties and directly participate in matrix remodeling (46). TGF-β remains the dominant molecular driver at this stage. Engagement of TβRI/II activates Smad2/3 signaling and induces expression of ECM-related genes such as COL1 and FN (47, 48), while suppressing MMP-2 and MMP-9. In addition, PI3K/Akt, JAK/STAT3, and Wnt/β-catenin pathways support fibroblast activation and sustain myofibroblast survival (49, 50).

Adaptive immune polarization reinforces this macrophage–fibroblast axis. Th2 cells produce IL-4 and IL-13, which promote macrophage polarization toward profibrotic states and indirectly enhance fibroblast activation (51). Th17 cells secrete IL-17A, driving fibroblast proliferation, myofibroblast differentiation, and pathological ECM accumulation (52). Regulatory T cells (Tregs), despite their immunosuppressive role, acquire profibrotic activity in fibrotic lungs under persistent TGF-β signaling (53–56). Notably, the reported functions of individual T cell subsets in pulmonary fibrosis are not fully concordant across studies, with both protective and pathogenic roles described. The molecular determinants that govern these divergent T cell programs, particularly in human disease, remain incompletely defined (57). B cells further modulate chronic fibrotic immunity. Activated B2 cells enhance cytokine production through NF-κB dependent release of IL-6 and TNF-α 112, whereas regulatory B cells exert stage-dependent immunomodulatory effects through IL-10, IL-35, and PD-L1 mediated suppression of effector T cells (58–60).

Emerging evidence further indicates that metabolic reprogramming of immune cells contributes to fibrotic progression. Profibrotic macrophages preferentially rely on oxidative metabolism and fatty acid oxidation, whereas pro-inflammatory states are more closely associated with glycolytic programs (61, 62). Such immunometabolic shifts influence cytokine production, survival, and profibrotic effector functions, adding an additional regulatory layer to immune–fibroblast interactions. In parallel, cytotoxic immune surveillance progressively weakens during fibrogenesis. Chronic exposure to TGF-β and IL-10 drives NK cell exhaustion with reduced IFN-γ production (63, 64) and CD8^+^ T cells undergo a similar exhaustion program through activation of the PD-1/PD-L1 axis, attenuating STAT1-dependent antifibrotic responses (58, 65, 66). Loss of effective immune-mediated clearance permits survival and accumulation of activated myofibroblasts. Together, these processes establish a self-reinforcing immune–fibroblast loop that sustains fibroblast activation, myofibroblast persistence, and progressive ECM deposition, ultimately driving irreversible lung remodeling (**Table 1**). Importantly, the immune mechanisms described above do not operate uniformly across all macrophage and T cell populations. Depending on disease stage, tissue niche, and microenvironmental context, specific subsets may exert either antifibrotic or profibrotic activities (67, 68). Determining which immune states actively drive fibrogenesis versus those that represent secondary adaptations to tissue remodeling therefore remains an important unresolved issue.

**Table 1:** Immunological characteristics of key immune cells during different stages of pulmonary fibrosis progression.

Immune cell	Key cytokines/effectors	Acute phase	Fibrotic phase	References
Neutrophils	MMP-8, MMP-9, ROS, NETs, IL-8, TNF-α	Participate in pathogen clearance and removal of necrotic cells	Promote fibrogenesis through protease release and persistent inflammation	(29, 30, 142)
Macrophages	M1: TNF-α, IL-1β, IL-6, CXCL9, CXCL10, MMP-3, MMP-13; M2: IL-10, TGF-β, PDGF	Initiate inflammatory response (M1 polarization)	Mediate tissue remodeling and fibrosis (M2 polarization)	(35, 45, 143, 144)
DCs	TNF-α, IL-1β, MMPs, TIMPs	Amplify local inflammation through antigen presentation and cytokine release	May exert anti-fibrotic roles early; promote fibrosis at late stages	(39, 40, 145, 146)
Th1 Cells	IFN-γ, TNF-β	Potentiate inflammation and immune activation	May contribute to fibroblast activation and tissue damage	(41, 147, 148)
Th2 Cells	IL-4, IL-5, IL-13	—	Promote fibroblast proliferation and ECM production	(51)
Th17 Cells	IL-17A	Amplify neutrophilic inflammation	Drive chronic inflammation and fibrogenesis	(52)
Treg Cells	IL-10, TGF-β	Suppress excessive immune activation	May paradoxically promote fibrosis via TGF-β secretion	(149)
CD8^+^ T Cells	TNF-α, IFN-γ	Exert cytotoxic effects against infected or damaged cells	May suppress fibroblast accumulation and fibrogenesis	(42, 150)
Trm	IFN-γ	Sustain local immune responses	Associated with persistent inflammation and fibrosis	(58, 151)
B Cells	IL-6, IL-4, TNF-α	Enhance humoral and proinflammatory responses	Contribute to autoimmunity and fibrogenic signaling	(152)
NK Cells	IFN-γ, TNF-α	Suppress inflammation via cytotoxicity and cytokine modulation	—	(153, 154)

## 3 Immune changes in pulmonary fibrosis across diverse etiologies

Pulmonary fibrosis is invariably accompanied by immune dysregulation, but the immune pathways that dominate fibrotic progression vary across disease etiologies. Building on the immune states and regulatory circuits outlined in Section 2, this section considers different fibrotic disorders as reflecting distinct patterns of engagement of shared immunological mechanisms. Using disease–associated interstitial lung disease (CTD-ILD), IPF, post-viral pulmonary fibrosis (PV-PF) and chronic obstructive pulmonary disease–associated pulmonary fibrosis (COPD-PF) as representative examples, we integrate clinical and molecular observations to outline both common immune features and disease-specific biases. In this context, disease heterogeneity is interpreted as differential activation of core immune programs that converge on fibroblast activation, extracellular matrix accumulation, and tissue remodeling.

### 3.1 Connective tissue disease-associated interstitial lung disease

**Figure 2:**
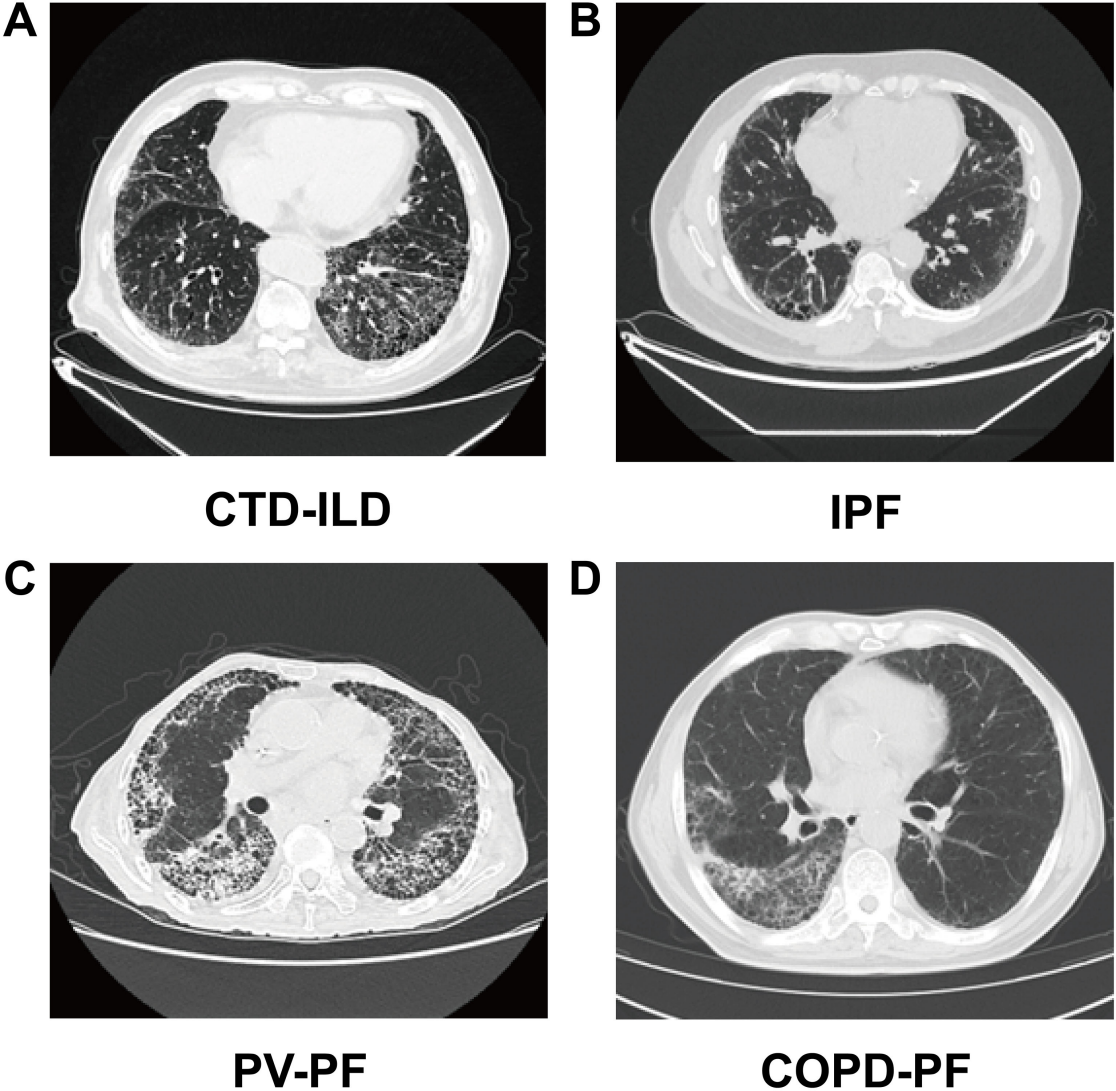
Representative high-resolution computed tomography (HRCT) images of fibrotic interstitial lung diseases (ILDs) with distinct etiologies. **(A)** connective tissue disease–associated interstitial lung disease (CTD-ILD): Nonspecific interstitial pneumonia (NSIP) pattern characterized by ground-glass opacities (GGO), reticulation, and traction bronchiectasis with symmetric involvement of the lower lobes. **(B)** idiopathic pulmonary fibrosis (IPF): Usual interstitial pneumonia (UIP) pattern showing subpleural honeycombing, traction bronchiectasis, and basal predominance. **(C)** post-viral pulmonary fibrosis (PV-PF): Pulmonary vasculopathy-associated pulmonary fibrosis with early-stage ground-glass opacities and crazy paving, progressing to reticulation, volume loss, and atypical fibrotic changes. **(D)** chronic obstructive pulmonary disease–associated pulmonary fibrosis (COPD-PF): Combined pulmonary fibrosis and emphysema with upper lobe–predominant emphysema and patchy, asymmetric fibrosis in the lower lobes.

CTD-ILD is a frequent and clinically significant complication of systemic rheumatic diseases such as systemic sclerosis (SSc), rheumatoid arthritis (RA), and dermatomyositis/polymyositis (DM/PM), contributing to substantial morbidity and mortality. Key pathological features include immune-mediated vasculitis and abnormal connective tissue remodeling involving multiple organs (69, 70). The lungs, due to their rich collagen matrix and vasculature, are especially vulnerable and often the earliest site of involvement (71). Disease progression is predominantly driven by sustained autoimmune-mediated immune disruption within the lung microenvironment. Autoantibodies targeting lung-specific antigens activate alveolar macrophages and impair endothelial function, triggering chronic inflammation, granulomatous lesions, and ECM remodeling (72). Functional shifts in innate and adaptive immune cells further perpetuate alveolar damage and interstitial fibrosis (73). Clinically, CTD-ILD presents with progressive dyspnea and persistent cough. Lung function tests show impaired diffusion capacity, while imaging typically reveals bilateral basal-predominant ground-glass opacities, reticulation, and honeycombing (74), reflecting the progression from inflammation to fibrosis (75)(**Figure 2A**).

Pulmonary injury in CTD stems from systemic immune dysregulation, involving both humoral and cellular pathways. This leads to persistent autoimmune activation within the pulmonary immune niche, a key driver of CTD-ILD. In SSc-ILD, dense B cell aggregates and ectopic follicles are found in lung tissue (76), in RA-ILD, coordinated CD4^+^ T and B cell infiltration is observed (77), highlighting B cells as central players. Beyond producing autoantibodies, B cells activate the CD40/CD40L axis, stimulating T cells and macrophages and inducing NF-κB–dependent IL-6 and TNF-α production (77). These cytokines promote fibroblast activation and collagen synthesis. Elevated IL-6 in bronchoalveolar lavage fluid (BALF) correlates with alveolar macrophage activation and predicts functional decline (DLCO, FVC) in SSc patients (53). T cells also shape fibrogenesis: Th2 cytokines (IL-4, IL-13) polarize M2 macrophages, driving remodeling—a mechanism confirmed in SSc-ILD samples and models (78). Additionally, Th17-derived IL-17 synergizes with TGF-β to activate fibroblasts and stimulate ECM deposition. JAK/STAT pathway activation amplifies this loop via IL-6 and IL-4, accelerating fibrosis (79).

Hyperactivation of both B and T cells underlies CTD-ILD pathogenesis. Elevated ANA, anti-Scl-70, and anti-Jo-1 autoantibodies serve not only as diagnostic markers but also as direct mediators of lung injury (80). These antibodies bind AECs and basement membrane antigens, forming immune complexes that activate complement in alveolar septa (81). This elicits local inflammation, cytotoxicity, and macrophage/dendritic cell activation, worsening epithelial damage and immune imbalance (82) Furthermore, autoantibody-driven activation of endothelial cells and fibroblasts upregulates chemokines and adhesion molecules (83), promoting immune cell recruitment and lesion expansion, which culminates in pathological ECM accumulation (84).

### 3.2 Idiopathic pulmonary fibrosis

IPF is a chronic, progressive interstitial lung disease strongly associated with aging, with both incidence and mortality rising with age. It displays hallmark features of cellular senescence, including telomere shortening, senescent cell accumulation, and mitochondrial dysfunction (85), which together create a vulnerable pulmonary microenvironment prone to injury and impaired repair (86). AEC II, which are central to alveolar regeneration, display profound senescence in IPF, with compromised functionality essential for lung repair (87). This is associated with disruption of the alveolar barrier, thereby facilitating fibroblast activation and phenotypic transition (88) (**Figure 2B**). The resulting expansion of myofibroblasts and ECM deposition leads to irreversible lung remodeling and functional decline. Clinically, IPF manifests as progressive dyspnea and dry cough. Pulmonary function tests show restrictive ventilatory defects and declining diffusing capacity (89). High-resolution computed tomography (HRCT) often reveals basal and subpleural reticulation, traction bronchiectasis, and honeycombing, reflecting transition from epithelial damage to fibrotic remodeling (90), in a setting characterized by aging-associated epithelial vulnerability and low-grade immune dysregulation.

IPF is not typically an overtly inflammatory disease, and sustained high-grade inflammation is not a defining clinical feature. Nevertheless, low-level immune activation is frequently detectable during progression and often co-localizes with epithelial injury and remodeling processes (91). This pattern is commonly interpreted as a downstream response to age-related tissue damage and defective epithelial repair rather than a primary initiating trigger (92). Clinical experience is broadly consistent with this view: non-selective immunosuppression has failed to provide benefit in IPF and, in some settings, has been associated with worse outcomes, arguing against inflammation control alone as an effective therapeutic strategy. Importantly, however, these negative results do not exclude a meaningful role for immune dysregulation. A more plausible interpretation is that immune pathways may contribute in a context- and stage-dependent manner—acting as modifiers of epithelial stress responses and fibroblast activation rather than sole disease drivers. Defining which immune programs are causal, which are adaptive, and which are merely correlative in human IPF therefore remains an open question and will require more refined, human-based mechanistic studies.

Recent experimental studies indicate that profibrotic DC activity can occur independently of conventional CD4^+^ T cell activation. In bleomycin models, DCs promote fibrosis by inducing IL-17 production from innate lymphoid populations, providing a potential explanation for IL-17–driven, steroid-insensitive fibrotic pathways (93). Innate immune alterations constitute a major component of the dysregulated IPF microenvironment. In this setting, macrophages preferentially acquire alternatively activated phenotypes and secrete profibrotic mediators such as TGF-β and PDGF, thereby directly promoting fibroblast activation and ECM accumulation (94). In parallel, DCs in fibrotic lungs exhibit functional reprogramming, with diminished antigen-presenting capacity but preserved or enhanced ability to shape local inflammatory and repair-associated signaling networks (95, 96). Lung Treg numbers rise, but their suppressive function is often impaired, compromising immune control (57). Imbalances among Th1, Th2, and Th17 subsets further contribute to a permissive profibrotic milieu. IL-17, enriched within fibrotic regions, amplifies TGF-β signaling and promotes fibroblast proliferation and matrix synthesis, reinforcing fibrotic progression (97). B cells, meanwhile, form ectopic follicle-like aggregates in fibrotic areas, acting as local germinal centers that sustain autoreactive responses (98). These B cell populations generate autoantibodies and enhance immune cell crosstalk, further stabilizing the fibrotic immune network (99). Age-associated mitochondrial dysfunction in immune cells exacerbates ROS production and disrupts immunometabolic homeostasis, impairing immune resolution and perpetuating chronic fibrotic signaling (95).

### 3.3 Post-viral pulmonary fibrosis

Acute viral infections often provoke intense immune activation and lung injury. In some individuals, inflammation persists post-infection and repair remains inadequate, leading to prolonged alveolar remodeling and ECM accumulation—hallmarks of PV-PF. This has been observed following SARS-CoV-2 (100), SARS, MERS, and severe influenza. PV-PF usually develops weeks to months after infection resolution, manifesting as dry cough, exertional dyspnea, and reduced DLCO. HRCT shows peripheral and basal ground-glass opacities, reticulation, and traction bronchiectasis; focal honeycombing may occur, indicating progression to fibrosis (101). These findings underscore persistent immune dysregulation in the context of unresolved epithelial injury as a central feature of fibrotic evolution after viral infection (**Figure 2C**).

Post-viral clearance, immune homeostasis often remains disturbed. Some immune cells persist in memory-like or activated states, secreting pro-inflammatory and profibrotic cytokines (IL-6, TNF-α, TGF-β), sustaining chronic inflammation (102). Alfaro et al. (103) reported elevated plasma TGF-β in COVID-19 convalescents, correlating with impaired lung function (103). In the early stages of infection, PAMPs activate macrophages via PRRs (e.g., TLR3, RIG-I), triggering cytokine storms with IL-1β, IL-6, TNF-α, and causing epithelial apoptosis and injury (104, 105). After viral clearance, macrophages polarize to M2 under IL-4/IL-10 influence, releasing TGF-β and PDGF, which promote fibroblast proliferation and collagen production. Abundant CD163^+^ M2 macrophages in severe COVID-19 lungs suggest their role in ongoing fibrotic remodeling (106).

Persistent alveolar damage further drives fibrosis. SARS-CoV-2 injures AECs and endothelium, releasing DAMPs and activating coagulation cascades that amplify inflammation (107). AEC II, critical for alveolar repair, are highly vulnerable to viral damage. Their impaired regeneration accelerates fibrosis. In murine influenza models, AEC II cells fail to differentiate into AEC I, a defect linked to hypoxia and elevated HIF-1α (108) leading to expansion of basal-like epithelial cells and disrupted alveolar architecture (109). These epithelial–fibroblast interaction defects are central to PV-PF pathogenesis, though human validation remains needed.

Adaptive immune remodeling also sustains inflammation and fibrosis in PV-PF. Increased Th17 cells in COVID-19 suggest persistent Th17 activation (110). IL-17A promotes neutrophil recruitment and stimulates fibroblasts to produce type I/III collagens (111). Treg/Th17 imbalance is key in PV-PF, with IL-17A and TGF-β synergistically enhancing fibrosis. Single-cell transcriptomics of BALF from COVID-19 convalescents revealed enriched CD8^+^ TEM cells in profibrotic phenotypes (112). These cells drive chronic antigen responses and enhance macrophage IL-1β production, impairing AEC II to AEC I transdifferentiation and alveolar repair (113).

PV-PF is therefore better viewed as a condition characterized by prolonged immune activation superimposed on epithelial dysfunction and impaired repair, rather than a simple post-viral sequela. Unlike other pulmonary fibrosis types, PV-PF features a mismatch between acute injury and chronic maladaptive repair, contributing to its clinical heterogeneity and unpredictable progression. This highlights the urgent need to clarify immune regulatory mechanisms to guide targeted immunotherapies.

### 3.4 COPD-associated pulmonary fibrosis

COPD is a common chronic respiratory disease characterized by persistent airway inflammation, airflow limitation, and irreversible lung damage. Unlike other pulmonary fibroses that mainly affect alveoli, COPD-PF is confined to airway walls and surrounding tissues, often accompanied by emphysema and focal damage, with slower progression (114). This heterogeneity arises from chronic exposure to harmful agents like smoking and pollution, which continuously activate local immune cells. Interactions among epithelial, immune, and stromal cells drive localized inflammation and fibrosis, causing structurally variable lesions. Clinically, patients present with chronic cough, sputum, and worsening dyspnea; advanced stages may involve respiratory failure or recurrent infections. Pulmonary function tests show reduced FEV_1_ and FEV_1_/FVC ratio indicating irreversible obstruction, sometimes with restrictive defects. HRCT reveals emphysema, small airway narrowing or obliteration, and peribronchovascular fibrosis; focal honeycombing is rare, and diffuse reticulation or traction bronchiectasis uncommon (115). These findings indicate that fibrosis in COPD is predominantly associated with chronic localized inflammation and airway-centered remodeling rather than diffuse alveolar injury (**Figure 2D**).

Early chronic exposure to particulates, gases, and infections damages alveolar and airway epithelium, releasing pro-inflammatory cytokines like IL-8 and TNF-α, which activate neutrophils and M1 macrophages to clear pathogens (116). These cells release elastases and MMPs (MMP-9, MMP-12), accelerating ECM breakdown and tissue injury, leading to airway remodeling and alveolar disruption. As COPD progresses, the immune microenvironment shifts towards repair and fibrosis, with increased M2 macrophages and persistent profibrotic mediators such as IL-13, IL-4, and TGF-β (117) activating fibroblasts and promoting collagen deposition in small airways (118).

Adaptive immune dysregulation also contributes importantly to COPD fibrosis. Increased activity of CD8^+^ and Th1 (CD4^+^) cells in COPD lungs promotes epithelial damage and remodeling via cytotoxic and pro-inflammatory mediators like IFN-γ and granzyme B (119). In certain COPD subsets, a shift towards Th2 polarization and impaired Treg function have been observed, suggesting that immune dysregulation and lineage plasticity drive distinct disease phenotypes and progression (120). Overall, the COPD immune microenvironment evolves from acute inflammation to chronic fibrosis, with local immune cell interactions driving disease heterogeneity. These interactions present potential targets for precision therapies.

**Figure 3:**
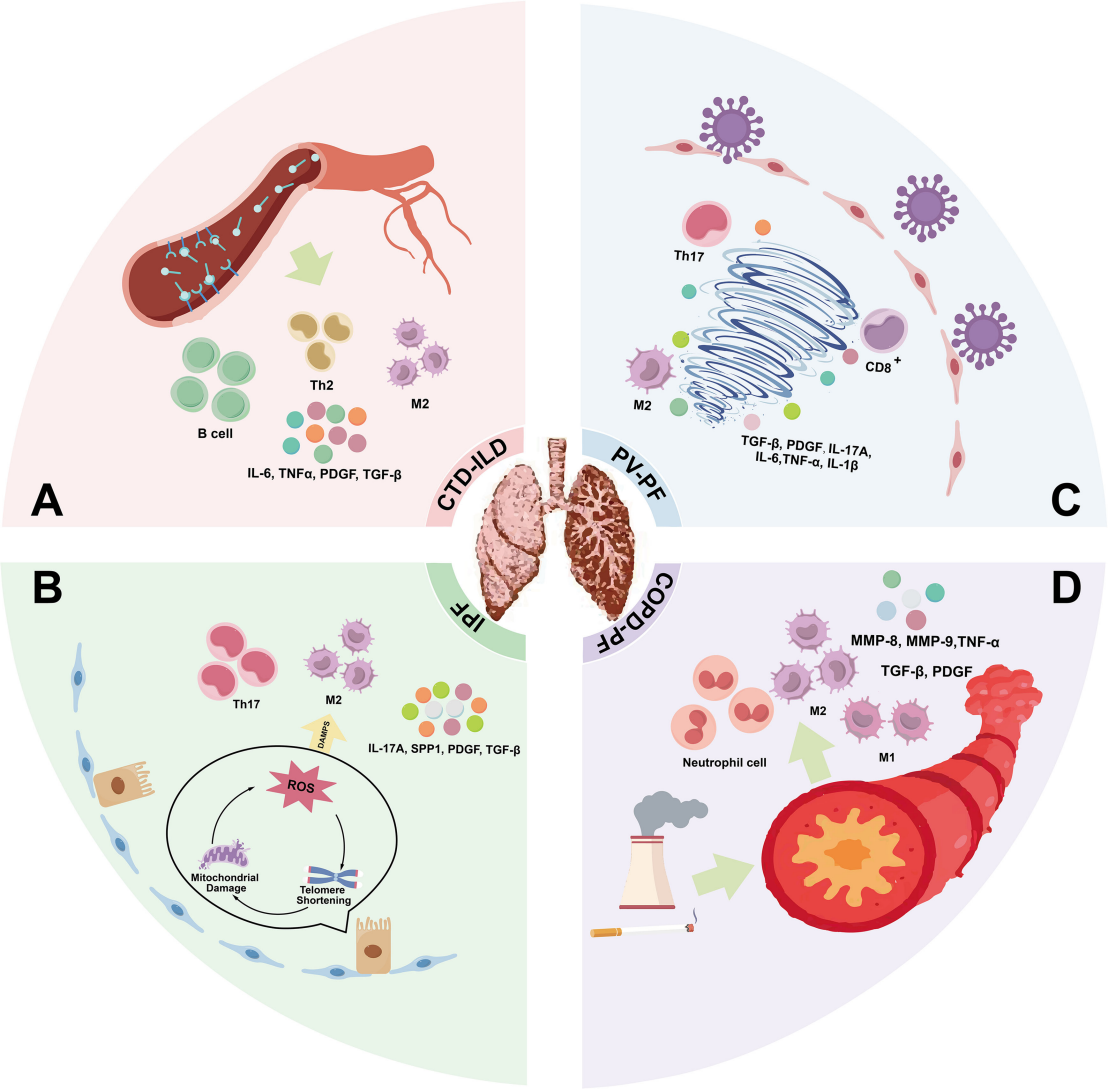
Distinct immune microenvironmental characteristics and profibrotic signaling profiles across etiologic subtypes of pulmonary fibrosis. **(A)** CTD-ILD: Autoantibody-mediated basement membrane injury and vascular damage lead to immune dysregulation characterized by B cell activation, Th2 cell polarization, and M2 macrophage skewing. Elevated levels of IL-6, TNF-α, PDGF, and TGF-β promote pulmonary inflammation and fibrogenesis. **(B)** IPF: Progressive AECs senescence, mitochondrial dysfunction, and ROS accumulation impair AEC II cell renewal and regeneration. Dysregulated immune profiles involving Th17 cells and M2 macrophages contribute to the release of TGF-β, PDGF, IL-17A, and secreted phosphoprotein 1 (SPP1), driving fibroblast activation, collagen deposition, and tissue remodeling. **(C)** PV-PF: Viral insult to alveolar epithelial cells triggers chronic low-grade inflammation. Aberrant immune activation involving Th17 cells, M2 macrophages, and CD8^+^ T cells induces a cytokine storm and stimulates profibrotic remodeling. **(D)** COPD-PF: Chronic airway exposure to smoking or dust leads to epithelial injury and structural scarring. Neutrophil-driven inflammation, along with MMP-8, MMP-9, TNF-α, TGF-β, and PDGF release, promotes tissue remodeling. M1 macrophages contribute to local destruction and fibrosis, while chronic immune cell activation in the inflamed milieu perpetuates fibrotic progression.

Pulmonary fibrosis shows marked etiological and phenotypic diversity, with immune dysregulation representing a common underlying component. Whether caused by autoimmunity, aging, incomplete viral clearance, or chronic exposures, these pathways converge on prolonged pulmonary immune imbalance. For example, CTD-ILD arises from aberrant autoimmunity causing hyperactive responses, while IPF is linked to aging-related immune dysfunction and atypical inflammation (**Table 2**). Viral infections, drugs, and environmental insults also trigger abnormal immune activation contributing to fibrosis. The spatial and functional dynamics of immune cells in the lung dictate the balance between damage and repair, influencing fibrosis outcomes (**Figure 3**). Thus, therapies targeting immune cell infiltration or modulating resident immune functions to restore balance hold promise in pulmonary fibrosis management (**Table 3**).

**Table 2:** Comparative clinical, radiographic, and functional features of pulmonary fibrosis with diverse etiologies.

Category	CTD-ILD	IPF	PV-PF	COPD-PF
Etiology	Autoimmune diseases (e.g., SSc, RA, PM/DM); immune activation drives fibrosis	AECs injury with aging, genetic risk	Post-viral immune dysregulation and failed epithelial repair (e.g., COVID-19, H1N1)	Chronic inflammation due to smoking or dust; airway injury extends to interstitium
Clinical Features	Cough and dyspnea with systemic symptoms (rash, arthritis, Raynaud’s)	Progressive exertional dyspnea, dry cough, possible digital clubbing	Persistent cough, dyspnea, and hypoxemia during recovery	Chronic cough and sputum, dyspnea; emphysema signs
Pulmonary Function	Restrictive pattern; ↓DLCO, variable FVC	Restrictive decline; progressive ↓FVC and ↓DLCO	↓DLCO; mild FVC decline in early phase	Mixed obstructive + restrictive; ↓DLCO
Fibrosis Onset Time	Gradual (months–years); may fluctuate with autoimmune activity	Insidious onset; classic fibrosis in 1–3 years	Within 4–12 weeks post-infection; some rapidly progress	Slowly progressive; develops after repeated exacerbations
Imaging Features	NSIP pattern: GGO, reticulation, traction bronchiectasis; symmetric lower lobe involvement	UIP pattern: subpleural honeycombing, traction bronchiectasis, basal predominance	Early GGO and crazy-paving; later reticulation, volume loss; atypical fibrosis	Upper lobe emphysema + lower lobe patchy fibrosis; asymmetric

**Table 3:** Etiology-specific microenvironmental alterations, immune features, and key cytokine profiles in pulmonary fibrosis.

Category	Unique microenvironmental alterations	Predominant immune features	Characteristic cytokine changes
CTD-ILD	- Vascular injury - Basement membrane disruption - Exposure of self-antigens	- Autoimmune-mediated tissue damage - Aberrant activation of B and T cells - Increased M2 macrophage polarization	- IL-6, TNF-α, TGF-β - Activation of TGF-β/JAK2–STAT signaling
IPF	- Accumulation of cellular senescence - Mitochondrial dysfunction - ROS accumulation	Dysfunction of AEC II - Increased M2 macrophages and impaired dendritic cell function - Imbalanced Th1/Th2/Th17 responses	- TGF-β, PDGF, IL-10, IL-17A - Presence of SPP1^+^ macrophages
PV-PF	- Disruption of immune–epithelial progenitor cell niche - Persistent low-grade inflammation	- Immune dysregulation following viral clearance - Cytokine storm with overactivation of Th1/Th17 responses - Increased M2 macrophage polarization	- HIF-1α - IL-6, TNF-α, TGF-β, IL-1β
COPD-PF	- Chronic airway inflammation - Focal tissue destruction	- Neutrophil-dominated inflammation - Active CD8^+^ T cell infiltration - Imbalance in protease–antiprotease activity - Increased M2 macrophage polarization	- IL-8, TNF-α, TGF-β - CD8^+^ T cell-derived cytokines

## 4 Immunomodulation and translational treatment strategies for pulmonary fibrosis

Drawing on the mechanistic and disease-specific evidence discussed above, immune-derived signals have been shown to regulate fibroblast activation and extracellular matrix remodeling in pulmonary fibrosis. However, the dominant immune programs that shape these processes differ across disease etiologies and disease stages. This heterogeneity helps explain why, despite clear involvement of immune pathways in fibrogenesis, broad and non-selective immunosuppressive therapies have shown limited efficacy and, in some settings, unfavorable outcomes. Collectively, these observations underscore the need for precise, mechanism-guided immunomodulation rather than generalized immune suppression. Accordingly, this section summarizes the immunoregulatory properties of currently approved antifibrotic agents, reviews emerging targeted immune-based therapies, and discusses future integrated strategies that combine disease control, antifibrotic intervention, and selective immune modulation.

### 4.1 Latent immunomodulatory effects of current antifibrotic agents

Fibroblasts are the main effectors in pulmonary fibrosis, making their pathological activation and ECM deposition key therapeutic targets. Approved antifibrotics—pirfenidone and nintedanib—work accordingly: pirfenidone reduces fibroblast activity and collagen synthesis, at least in part through modulation of profibrotic mediators such as TGF-β–related signaling, while nintedanib, a multitarget tyrosine kinase inhibitor, inhibits fibroblast proliferation and differentiation, limiting ECM accumulation (10). Beyond these direct effects, both drugs also modulate the immune microenvironment. Pirfenidone attenuates IL-4/IL-13–induced M2 macrophage polarization and reduces profibrotic markers such as TGF-β and ARG1 (and murine Ym1 in experimental systems) *in vitro*, decreasing M2 infiltration and TGF-β/Smad3 signaling in radiation-induced fibrosis models (11). Similarly, nintedanib lowers M2 markers (CD206, CD163, MerTK) and TGF-β production in macrophages from systemic sclerosis–associated ILD patients, thereby disrupting profibrotic macrophage–fibroblast interactions. In bleomycin models, it modulates macrophage phenotypes and macrophage-driven fibrogenic signaling, contributing to attenuation of fibrosis (121). Despite these immunomodulatory actions, their lack of selective targeting of defined immune circuits and limited efficacy restrict precise immune regulation and reversal of established fibrosis, contributing to suboptimal outcomes in advanced disease.

In connective tissue CTD-ILD, immunomodulatory and immunosuppressive agents, including glucocorticoids, cyclophosphamide, azathioprine, and mycophenolate mofetil, are widely used to mitigate immune activation and inflammatory injury, particularly in inflammation-predominant phenotypes or in the setting of rapid clinical deterioration. However, the strength of evidence supporting individual agents varies across CTD-ILD subtypes, and these therapies are not uniformly established as the universal first-line option for all immune-mediated fibrotic lung diseases (122–124). By contrast, early clinical efforts employing broad immunosuppressive strategies in IPF including corticosteroids, azathioprine, and cyclophosphamide, as well as cytokine-targeted therapies such as interferon (IFN)-γ and anti–tumor necrosis factor agents—have largely failed to demonstrate clinical benefit and, in some cases, have worsened outcomes. These disappointing results indicate a fundamental mismatch between conventional immunosuppressive approaches and IPF disease biology. Because profibrotic immune signaling in IPF is dominated by macrophage–fibroblast and epithelial–fibroblast circuits rather than classical CD4^+^ T cell–centered inflammation, suppression of T cell–focused pathways alone is unlikely to effectively disrupt fibrogenic signaling and may partially explain the limited responsiveness to corticosteroid-based immunosuppression (125). Moreover, non-selective immune suppression compromises innate immune defenses, increases susceptibility to infection, and may further aggravate established fibrotic pathology. Collectively, these negative trials highlight that global suppression of immune activity is insufficient and potentially detrimental, and instead underscore the necessity to identify discrete immune circuits that mechanistically couple inflammatory signaling to fibroblast activation, tissue remodeling, and progressive fibrosis (126).

Together, these observations suggest that the limited efficacy of current therapies in fibrotic lung disease reflects a combination of suboptimal target selection, treatment at advanced stages, and dependence on non-selective immune suppression. The repeated failure of immune-targeted interventions further suggests that many immune abnormalities observed in fibrotic lungs may be secondary or adaptive rather than primary drivers of disease. Distinguishing immune pathways that are mechanistically linked to fibrogenesis from those representing bystander alterations therefore remains a major challenge for the development of precise immunomodulatory therapies. This subsection illustrates how both antifibrotic agents and conventional immunosuppressants expose the limitations of non-selective immune modulation and support a shift toward mechanism-guided strategies.

### 4.2 Microenvironment—targeted precision therapy for multietiological pulmonary fibrosis

Across etiologies, emerging immunomodulatory strategies can be broadly grouped into approaches targeting humoral immunity, macrophage polarization, checkpoint signaling, and innate inflammatory danger pathways. Traditional pharmacotherapies partially intersect with immune–fibrosis crosstalk, but their broad mechanisms of action and limited pathway selectivity hinder precise modulation of disease-driving immune programs and rarely permit reversal of established fibrotic architecture. With growing insight into the immunopathogenesis of pulmonary fibrosis, therapeutic development has increasingly shifted toward strategies that target pathogenic immune states and their functional coupling to fibroblast activation within the tissue microenvironment. Across distinct etiological subtypes—including CTD-ILD, IPF, virus-associated, and COPD-related pulmonary fibrosis—emerging immunomodulatory agents have entered preclinical and early clinical evaluation, reflecting an evolving paradigm in which discrete immune pathways, rather than global immune suppression, are selectively manipulated to achieve disease modification.

In CTD-ILD, aberrant humoral immunity, autoantibody production, and ectopic lymphoid organization represent prominent immunopathogenic features. Dysregulated B cell activation and survival sustain immune activation and amplify profibrotic cytokine networks, thereby facilitating fibroblast activation and matrix deposition. Against this background, therapeutic efforts have largely focused on modulating B cell–driven immune circuits. Blockade of the IL-6 receptor with tocilizumab has been shown to preserve lung function in patients with early-stage SSc-ILD, supporting its use in selected CTD-ILD populations (12). Direct B cell depletion with rituximab reduces autoantibody production and dampens immune activation, and randomized trials together with real-world cohort studies in CTD-ILD and rheumatoid arthritis–associated ILD report stabilization of pulmonary function with acceptable safety profiles, supporting B cell–targeted therapy as a feasible clinical option (127, 128). More recently, immune reconstitution approaches using CD19-targeting chimeric antigen receptor (CAR) T cells have been reported in severe systemic sclerosis, demonstrating the capacity to profoundly reshape pathogenic humoral immunity. In parallel, B cell–directed CAR-T therapy is being explored across a range of immune-mediated diseases, indicating a broader therapeutic interest in deep B cell immune modulation. By contrast, targeting upstream B cell signaling kinases remains supported mainly by preclinical evidence. BTK, a central mediator of B cell receptor signaling, has been implicated in experimental systemic sclerosis–associated fibrosis, and BTK inhibitors such as BTKB66 attenuate inflammatory infiltration and tissue fibrosis in murine models (13), although clinical efficacy data in CTD-ILD are still limited.

Although IPF lacks prominent immune lineage abnormalities, its microenvironment features dysregulated CD4^+^ T cells, sustained M2 macrophage activation, and immune-metabolic imbalance. Th17 cells and their cytokine IL-17A drive chronic inflammation and fibrosis by enhancing TGF-β signaling, promoting fibroblast proliferation and ECM deposition—the so-called “Th17/IL-17 axis.” However, translation of immune-targeted strategies into effective IPF therapies has remained limited, and broadly acting immunomodulatory approaches have not demonstrated consistent disease-modifying benefit; recent clinical development has therefore increasingly shifted toward mechanism-guided, pathway-specific interventions. In this context, Nerandomilast (Jascayd), a selective PDE4B inhibitor that modulates macrophage and monocyte-driven inflammatory signaling within the immune microenvironment, was approved by the U.S. FDA for idiopathic pulmonary fibrosis and progressive pulmonary fibrosis based on phase 3 evidence showing a smaller decline in FVC versus placebo (129). This finding supports the concept that modulation of macrophage- and monocyte-derived inflammatory signaling can indirectly attenuate fibroblast activation. Celada et al. showed that PD-1 deletion or PD-L1 blockade in bleomycin-induced fibrosis reduced lung inflammation, collagen accumulation, and expression of TGF-β and IL-17A, decreasing fibroblast collagen synthesis and highlighting PD-1/PD-L1 as a key profibrotic pathway (130). Meanwhile, M2 macrophages expressing CD206 contribute to fibrosis via profibrotic cytokines such as TGF-β and IL-6. Ghebremedhin et al. developed RP-832c, a CD206-targeting peptide that reduced fibrosis markers, inflammatory cytokines, and alveolar damage in mice and synergized with pirfenidone to enhance antifibrotic effects, underscoring its potential as an immunomodulatory therapy for IPF (14).

Virus-associated pulmonary fibrosis is shaped predominantly by excessive innate immune activation and unresolved inflammatory signaling following acute infection. Persistent activation of the IL-1β/NLRP3 inflammasome, aberrant JAK–STAT signaling, and sustained Th1/Th17 responses contribute to ongoing epithelial injury, fibroblast activation, and progressive ECM deposition. Within this context, targeted modulation of inflammatory danger-sensing pathways has attracted increasing attention. The IL-1 pathway occupies a central position in infection-associated lung injury, and treatment with anakinra, an IL-1 receptor antagonist, has been reported to improve respiratory function and facilitate resolution of fibrotic lesions in COVID-19 patients with refractory hypoxemia, demonstrating safety and potential antifibrotic benefit (131). Dysregulated JAK/STAT signaling also participates in post-viral fibrotic remodeling. Ruxolitinib, a selective JAK1/2 inhibitor approved for myelofibrosis, suppresses IL-6/JAK/STAT3–driven hyperinflammation in COVID-19, and randomized phase II studies indicate improvements in pulmonary function and inflammatory indices in severe disease (132). Additional clinical observations further support a role for JAK1/2 inhibition in mitigating virus-induced pulmonary fibrosis (133, 134).

In COPD-related pulmonary fibrosis, chronic innate immune–driven inflammation predominates, with neutrophil recruitment and sustained activation of NF-κB–dependent transcriptional programs shaping a profibrotic microenvironment. Excessive neutrophil chemotaxis promotes release of proteases and inflammatory mediators, thereby driving fibroblast activation and small-airway remodeling. CXCR2, a key receptor regulating neutrophil trafficking, is chronically activated in COPD; pharmacologic blockade with danirixin reduces neutrophil infiltration but produces modest clinical benefit, with higher doses associated with increased pneumonia risk, underscoring the need for cautious therapeutic calibration (135). The NF-κB pathway represents another convergence point linking inflammation to fibroblast activation. Parthenolide has been shown to reduce collagen deposition and inflammatory cell infiltration through inhibition of the NF-κB/Snail axis in experimental fibrosis models, supporting its potential relevance to COPD-associated fibrotic remodeling despite limited disease-specific validation (136). In addition, aberrant MMP activity contributes to airway remodeling and elastic fiber degradation in COPD, with elevated MMP-9/TIMP-1 ratios correlating with lung function decline (137). MMP inhibitors such as AS111793 and MMP-408 have demonstrated efficacy in attenuating inflammation and improving lung pathology in murine COPD models (138, 139), suggesting that modulation of protease-driven tissue remodeling may complement immune-targeted strategies. Notably, direct clinical evidence demonstrating that immunomodulatory interventions alter fibrotic outcomes in COPD-associated pulmonary fibrosis remains scarce. Future therapeutic development will likely require phenotype-based patient stratification. Rational combination strategies targeting inflammation, protease activity, and fibroblast activation may be necessary.

Beyond well-defined fibrotic subtypes, a subset of patients presents with nonspecific or unclassifiable ILD and atypical immune phenotypes, in whom overt immune cell abnormalities are often absent. The underlying immune drivers in these cases remain poorly resolved, limiting the applicability of single-target therapeutic approaches. Advancing precision therapy for this population will require integrative stratification that incorporates immune cell states, pathway activity, and etiological context. Rapid progress in single-cell and spatial transcriptomic technologies is progressively refining the cellular architecture of the fibrotic lung, thereby providing a foundation for aligning mechanistic insights with rational target selection and personalized intervention strategies (**Table 4**).

**Table 4:** Immunologically targeted therapeutics in pulmonary fibrosis across etiologies.

Drug	Target	Experimental/clinical context	Therapeutic outcome	Reference
Tocilizumab	IL-6 receptor antagonist	Clinical trial in SSc-ILD	Improved pulmonary function; FVC and DLCO stabilized or increased	(12)
Rituximab	CD20^+^ B cell depletion	Clinical application and follow-up in CTD-ILD	Reduced autoantibody production and pathological immune response; improved FVC	(12)
Anti-PD-L1 antibody	Suppresses IL-17A/TGF-β axis	IPF (human samples and bleomycin-induced mouse model)	Significant reduction in inflammation and collagen deposition in lung tissue	(130)
RP-832c	Targets CD206^+^ M2 macrophages	Bleomycin-induced pulmonary fibrosis in mice	Decreased lung collagen accumulation and lower Ashcroft scores	(14)
Anakinra	IL-1 receptor antagonist	Late-stage COVID-19 patients with fibrotic lesions	CT imaging showed regression or reduction of fibrotic areas	(131)
Ruxolitinib	Inhibits IL-6/JAK/STAT3 signaling pathway	COVID-19-related pulmonary fibrosis in human subjects	Significant improvement in lung function and oxygenation indices	(132–134)
Danirixin	CXCR2 antagonist	COPD-PF model	Attenuated pulmonary inflammation	(135)
Parthenolide	NF-κB pathway inhibitor	Bleomycin-induced pulmonary fibrosis in mice	Markedly reduced collagen deposition and inflammatory infiltration	(136)
AS111793	Selective MMP-12 inhibitor	Cigarette smoke-induced emphysema model in mice	Alleviated emphysematous changes and partially reversed lung structural damage	(138, 139)

### 4.3 Therapeutic approaches targeting etiology and immune regulation

Pulmonary fibrosis is currently managed primarily with etiology-directed therapy when available, anti-inflammatory or immunosuppressive agents in selected immune-mediated settings, and antifibrotic drugs that slow disease progression. In clinical practice, treatments such as glucocorticoids and conventional immunosuppressants are commonly used in inflammation-predominant or autoimmune-associated interstitial lung diseases, whereas pirfenidone and nintedanib remain the backbone therapies for idiopathic pulmonary fibrosis and other progressive fibrosing phenotypes. Despite these approaches, treatment responses are variable and disease progression often continues. Against this background, increasing attention has turned toward strategies that incorporate immune modulation alongside existing therapeutic backbones, with the goal of more precisely intervening in pathogenic immune processes that contribute to fibrotic progression.

The modulation of the immune microenvironment can be strategically implemented through a tiered intervention framework that systematically targets critical profibrotic mechanisms at three interrelated levels: etiological context, immune cell populations, and intracellular signaling pathways. At the cellular level, this framework emphasizes precise identification and selective targeting of key immune populations, with an emphasis on delineating their functional states and therapeutic windows for reversibility. At the molecular level, comprehensive elucidation of shared profibrotic signaling cascades—including TGF-β/Smad, and JAK/STAT pathways—is crucial to disrupt the positive feedback loops that potentiate the immune–fibroblast crosstalk from its inception. Finally, integrating patient-specific etiologies with immune phenotypic profiles enables the formulation of personalized, multi-targeted combination therapies, embodying the principle of immune stratification coupled with targeted synergy. This stratified intervention model not only deepens the systematic understanding of pulmonary fibrosis immunopathogenesis but also offers a conceptual framework to guide clinical translation. Recent advances in single-cell and spatially resolved transcriptomic technologies are beginning to accelerate this stratification paradigm by enabling high-resolution mapping of immune and stromal cell states, their functional programs, and their spatial organization within fibrotic niches (140, 141). These approaches have uncovered previously unrecognized profibrotic macrophage subsets, context-dependent T cell states, and spatially coupled immune–fibroblast interactions in human fibrotic lungs, thereby informing target prioritization and patient endotyping. Integration of such multidimensional datasets with clinical phenotypes is expected to further refine immune-based classification schemes and guide the development of precision immunomodulatory combination therapies. Through precise immune subtype delineation and identification of target-responsive intervals, clinicians may be able to rationally design combination regimens to enhance therapeutic efficacy while minimizing nonspecific immunosuppression, adverse events, and resource expenditure, ultimately improving patient outcomes and quality of life.

Many immune mechanisms and putative therapeutic targets discussed above have been defined mainly through preclinical studies, most often using bleomycin-induced models of lung fibrosis. These models have yielded important insights into immune–stromal interactions during fibrotic remodeling, but they only partially represent the clinical and pathological complexity of human pulmonary fibrosis. Bleomycin-induced fibrosis is initiated by acute epithelial injury with marked inflammatory responses and commonly follows a self-limited course, whereas human pulmonary fibrosis is typically chronic, frequently age-associated, and characterized by persistent epithelial dysfunction and progressive architectural distortion. Consequently, immune pathways that appear prominent in experimental settings may not exert comparable functions in human disease, and therapeutic effects observed in animal models should be interpreted cautiously. In addition, interspecies differences in immune cell composition, activation thresholds, and cytokine signaling further limit direct extrapolation from murine systems to humans. Mechanistic observations from animal studies should therefore be regarded primarily as hypothesis-generating and, whenever possible, corroborated using human lung tissues, patient-derived cellular systems, single-cell or spatial transcriptomic datasets, and well-characterized clinical cohorts to distinguish conserved drivers of fibrosis from context-dependent features of experimental injury models.

## 5 Prospect

Pulmonary fibrosis represents a common end stage of diverse chronic lung injuries and arises not only from excessive extracellular matrix deposition but also from dysregulation of multiple cellular populations and signaling networks. Although myofibroblasts constitute the principal effector cells of fibrosis, the immune microenvironment is highly heterogeneous and dynamically regulated. As summarized in this review, macrophages, dendritic cells, T cells, B cells, and NK cells participate in stage-dependent immune programs spanning immune homeostasis, inflammatory injury, and fibrotic remodeling, collectively shaping profibrotic signaling across etiologies. However, many proposed immune mechanisms have been defined primarily in animal models and remain insufficiently validated in human disease, limiting their translational relevance. In addition, while immune pathways described in hepatic and renal fibrosis provide useful conceptual reference points, their tissue-specific relevance in the lung requires systematic evaluation in human tissues and patient-derived systems.

Immune dysregulation is tightly linked to pulmonary fibrogenesis and gives rise to distinct immune patterns across disease contexts, complicating the development of uniform therapeutic strategies. Future studies will benefit from integrating multi-omics profiling, including single-cell and spatially resolved approaches, with detailed clinical phenotyping to establish models linking etiological subtypes, immune cell states, and dominant signaling programs. High-resolution spatial and temporal characterization of immune–fibroblast interactions will be particularly important for defining stage-specific immune states and their transitions during disease progression. Such information may clarify when and where specific immune pathways become pathogenic and inform the rational timing of immunomodulatory interventions. In parallel, longitudinal monitoring of immune cell subsets, profibrotic mediators, and pathway activity could enable dynamic assessment of disease evolution and therapeutic response, thereby supporting individualized treatment strategies and improved prognostic stratification.

**Figure 4:**
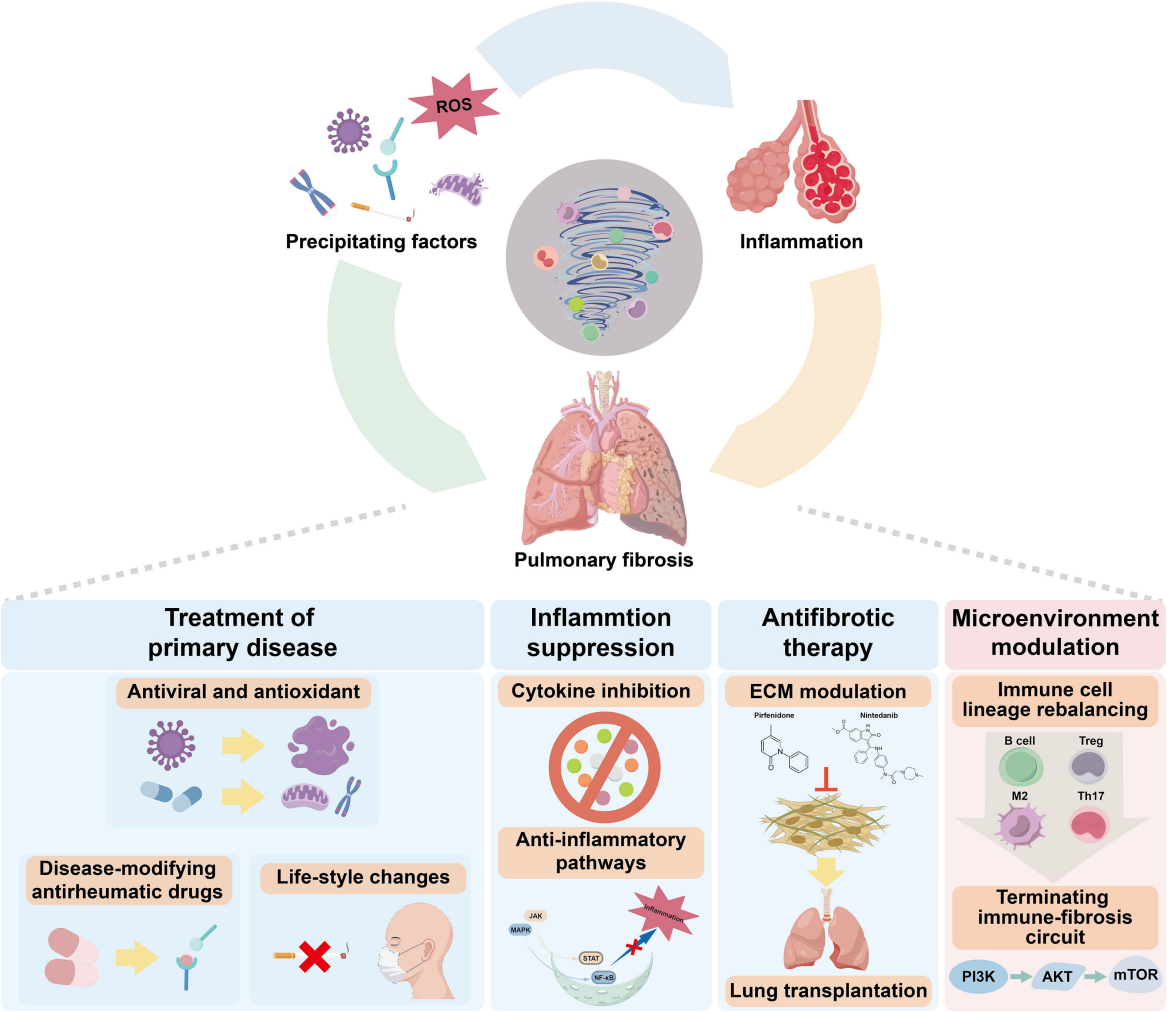
Therapeutic strategies targeting the pathogenesis and progression of pulmonary fibrosis. Pulmonary fibrosis can be initiated by various precipitating factors, including infections, autoimmune responses, and ROS, which either provoke inflammation or directly trigger fibrotic changes. Sustained inflammation further drives fibrotic remodeling. Current therapeutic strategies aim to interrupt disease progression at different stages: Treatment of the underlying disease includes antiviral and antioxidant therapies, disease-modifying antirheumatic drugs (DMARDs), and lifestyle modifications to eliminate causative factors; Suppression of inflammation involves targeting pro-inflammatory cytokines and signaling pathways (e.g., JAK/STAT, MAPK) to mitigate chronic inflammation and reduce fibrotic potential; Antifibrotic therapy seeks to regulate ECM production through agents such as pirfenidone and nintedanib. In cases of end-stage disease, lung transplantation may be necessary; Modulation of the tissue microenvironment aims to restore immune balance by reprogramming immune cell lineages (e.g., B cells, Tregs, M2 macrophages) and disrupting the self-sustaining immune-fibrosis loop through key signaling pathways such as PI3K–AKT–mTOR.

Current therapeutic options underscore both the limitations of existing approaches and the potential of more selective immune-based interventions. Although antifibrotic agents such as pirfenidone and nintedanib slow disease progression, they do not directly address the complexity of immune dysregulation underlying pulmonary fibrosis. Advances in understanding the immune microenvironment have facilitated the development of therapies targeting defined immunoregulatory pathways, several of which have shown encouraging activity in preclinical models and early clinical studies. Nevertheless, their long-term efficacy, safety, and generalizability across patient populations remain to be established. Future efforts should therefore prioritize the development of immunomodulatory strategies with greater target specificity and suitability for chronic administration, ideally integrated with antifibrotic therapy and etiological management, to improve long-term outcomes in pulmonary fibrosis (**Figure 4**).
